# Blood-Borne Markers of Fatigue in Competitive Athletes – Results from Simulated Training Camps

**DOI:** 10.1371/journal.pone.0148810

**Published:** 2016-02-18

**Authors:** Anne Hecksteden, Sabrina Skorski, Sascha Schwindling, Daniel Hammes, Mark Pfeiffer, Michael Kellmann, Alexander Ferrauti, Tim Meyer

**Affiliations:** 1 Institute of Sports and Preventive Medicine, Saarland University, Saarbruecken, Germany; 2 Institute of Sports Science, Johannes-Gutenberg University, Mainz, Germany; 3 Faculty of Sports Science, Ruhr-University of Bochum, Bochum, Germany; 4 Schools of Human Movement Studies and Psychology, The University of Queensland, Queensland, Australia; Universidad Europea de Madrid, SPAIN

## Abstract

Assessing current fatigue of athletes to fine-tune training prescriptions is a critical task in competitive sports. Blood-borne surrogate markers are widely used despite the scarcity of validation trials with representative subjects and interventions. Moreover, differences between training modes and disciplines (e.g. due to differences in eccentric force production or calorie turnover) have rarely been studied within a consistent design. Therefore, we investigated blood-borne fatigue markers during and after discipline-specific simulated training camps. A comprehensive panel of blood-born indicators was measured in 73 competitive athletes (28 cyclists, 22 team sports, 23 strength) at 3 time-points: after a run-in resting phase (d 1), after a 6-day induction of fatigue (d 8) and following a subsequent 2-day recovery period (d 11). Venous blood samples were collected between 8 and 10 a.m. Courses of blood-borne indicators are considered as fatigue dependent if a significant deviation from baseline is present at day 8 (Δfatigue) which significantly regresses towards baseline until day 11 (Δrecovery). With cycling, a fatigue dependent course was observed for creatine kinase (CK; Δfatigue 54±84 U/l; Δrecovery -60±83 U/l), urea (Δfatigue 11±9 mg/dl; Δrecovery -10±10 mg/dl), free testosterone (Δfatigue -1.3±2.1 pg/ml; Δrecovery 0.8±1.5 pg/ml) and insulin linke growth factor 1 (IGF-1; Δfatigue -56±28 ng/ml; Δrecovery 53±29 ng/ml). For urea and IGF-1 95% confidence intervals for days 1 and 11 did not overlap with day 8. With strength and high-intensity interval training, respectively, fatigue-dependent courses and separated 95% confidence intervals were present for CK (strength: Δfatigue 582±649 U/l; Δrecovery -618±419 U/l; HIIT: Δfatigue 863±952 U/l; Δrecovery -741±842 U/l) only. These results indicate that, within a comprehensive panel of blood-borne markers, changes in fatigue are most accurately reflected by urea and IGF-1 for cycling and by CK for strength training and team sport players.

## Introduction

Training load and competition frequency of elite athletes are steadily increasing and performance differences between race winners and “also-ran” are becoming more and more marginal. Consequently, athletes and coaches face the difficult task of maximizing training load and adaptation whilst avoiding insufficient recovery, which would lead to maladaptation, loss of performance and possibly to non-functional overreaching or the overtraining syndrome. In this situation, assessing the current fatigue status of an individual athlete is a critical task in order to fine-tune training prescriptions. The pivotal and most relevant objective characteristic of fatigue in competitive athletes is a decline in discipline specific performance [1]. However, requiring maximum effort, this parameter is not suitable for repeated routine assessment. Therefore, other indicators of fatigue and recovery have been investigated including a wide range of blood-borne parameters (biochemical [2,3,4], hormonal [2,4,5], immunological [6,7], psychological questionnaires [8] and the assessment of autonomous nervous system balance (heart rate and heart rate variability) [2,9,10].

Blood-borne parameters are particularly attractive surrogate markers for training-induced fatigue because of a minimal interference with the training process, high accuracy and precision of measurements and, in most cases, a clear physiological concept concerning their connection with exercise and fatigue [4]. However, so far no parameter could be established which represents changes in fatigue and recovery during athletic training cycles with adequate sensitivity and reproducibility [2,5,11]. This may partly be due to the scarcity in experimental trials with carefully standardized, fatiguing training interventions in already well-trained athletes. Therefore, the present study aims to describe changes in a panel of blood-borne indicators of fatigue and recovery during a simulated training camp in competitive athletes. Three disciplines were chosen to represent major training modes: endurance training (cycling), strength training, and high intensity interval training (ballgame / team sports). In addition to assessing the courses of the chosen blood-borne surrogate markers during and after a period of overload training, their changes will be compared to the expected decline and restoration of discipline-specific performance.

## Material and Methods

### General design

**Fig 1:**
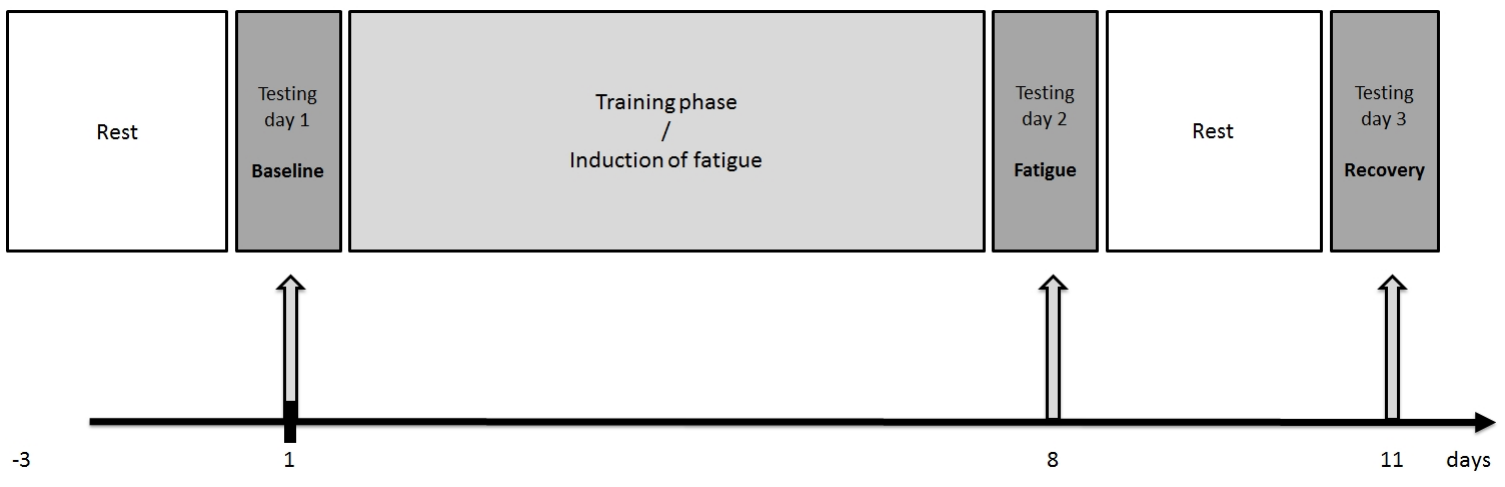
Design.

The study was a prospective, short-term training trial with follow-up testing. Tests were conducted at baseline following two days of rest (rested; d 1), after induction of fatigue by a discipline specific, six day strenuous training program (fatigue; d 8) as well as following an additional two day rest period (recovery, d 11). An overview of the general design is given in Fig 1. At testing days discipline specific performance was assessed to verify effective, reversible induction of fatigue. Preceding the exercise tests venous blood samples were collected from the antecubital vein by standard techniques to enable determination of blood-born indicators. Nutritional diaries were kept throughout the study period.

All tests were conducted in a University department (Cyclists: Saarland University, Germany; Strength athletes and team sports players: Ruhr Universität Bochum, Germany). For cyclists and ballgame players, tests were conducted during the respective preparation periods (cyclists: winter; ballgame players: summer). Strength athletes, not involved in competitions, were also studied during the summer season. The present investigation was carried out in accordance with the declaration of Helsinki and approved by the local ethics committee (Ärztekammer des Saarlandes, ID 46/13). All participants provided written informed consent prior to participation. Data are provided as supplemental material (S1 PDF).

### Subjects

Competitive cyclists (representing high-volume endurance training, n = 28), team sport players (representing intermittent, high-intensity load; n = 22), and strength athletes (n = 23) were studied. All athletes were examined during their respective preparatory season. For cyclists only holders of a national competition license were eligible for this trial. Analogously, ballgame players were eligible if they participated in a regional league organized by the respective federation. No competition level was required for strength athletes.

### Outcome measures

Blood-born parameters were included in the lab panel if they fulfilled the following criteria: 1) Evidence for an association with exercise training related fatigue. 2) Existing physiological insights into the mechanisms underlying this relationship. 3) Practicable sampling and analytical methodology, potentially enabling implementation on a routine basis. The resulting panel of blood-born indicators includes standard (creatine kinase (CK), urea, free-testosterone, c-reactive protein (CRP), cortisol [2,5,11]) as well as more recently proposed or less widespread parameters (adrenocorticotropic hormone (ACTH), glutamine (Gln), glutamate (Glu), insulin like growth factor 1 (IGF-1), IGF-1 binding protein 3 (IGF-BP3), tumor necrosis factor (TNF), interleukin 6 (IL-6), human growth hormone (HGH) [2,4]). The respective physiological domains span the major physical aspects of exercise training induced fatigue: metabolic status and anabolic/catabolic balance (urea, free-testosterone/cortisol ratio, IGF-1, IGF-BP3, IL-6, Gln, Glu,), neurohumoral regulation (ACTH, cortisol, ACTH/cortisol ratio, free-testosterone, HGH, IGF-1) muscle damage (CK) and inflammation (TNF, IL-6, CRP).

### Blood sampling

Venous blood samples were collected at the beginning of testing days. After reporting to the laboratory at a standardized time (between 8 and 10 a.m., intraindividually same hour for all tests) subjects rested in the supine position for 10 min prior to blood collection. A winged cannula was inserted into the antecubital vein during a short stasis (max. 30 sec.). Samples were processed immediately. Serum aliquots were frozen at -80°C within 60 min from blood collection and stored for later analysis.

### Analysis of blood samples

Blood count was performed immediately after sampling using an AcT 5 diff AL (Beckmann Coulter GmbH, Krefeld, Germany). CK, urea and CRP were analysed within 60 min by automated routine techniques (UniCell DxC 600 Synchron; Beckmann Coulter GmbH, Krefeld, Germany). Free-testosterone, IGF-1, IGF-BP3, TNF and ACTH were analysed in one run from frozen serum samples using commercially available ELISA kits (Free-testosterone: LDN Labor Diagnostika Nord GMBH & Co KG, Nordhorn, Germany; IGF-1 and IGF-BP3: mediagnost GmbH, Reutlingen, Germany; TNF: Immunotech, Reutlingen, Germany; ACTH: JBL International, Hamburg, Germany; all purchased via Beckmann Coulter GmbH, Krefeld, Germany). The respective protocols supplied by the manufacturer were meticulously followed. Each sample was analysed in double and the mean of the two measurements was used as estimate of sample concentration. Gln and Glu were determined by an accredited commercial laboratory using immunochemical methods (Studienzentrum, MVZ Labor Limbach, Heidelberg, Germany).

### Exercise testing

Stepwise, incremental exercise tests to exhaustion were conducted in all athletes to estimate maximum aerobic capacity and enable comparative characterization of subgroups. Discipline specific performance was assessed on all testing days to verify effective induction of a reversible increase in fatigue. An overview over testing procedures and parameters is given in Table 1. A detailed description of the employed methods is provided as supplemental material (S1 Text).

**Table 1:** Assessment of discipline specific performance (overview).

	Cycling	HIIT	Strength
Test	Cycling time trial (♂ 40km¸♀ 20km)—Own bike fixed on ergometer	Repeated sprint test on a non-motorized treadmill (6x4 s; 20 s rest)	Half squat and bench press
Criterion parameter	Overall performance time [s]	Mean peak velocity [m*s^-1^]	Maximal voluntary isometric contraction force (MVIC; [N]) Mean of the two exercises

Please compare supplemental material (S1 Text) for reproducible details.

### Induction of fatigue

**Fig 2:**
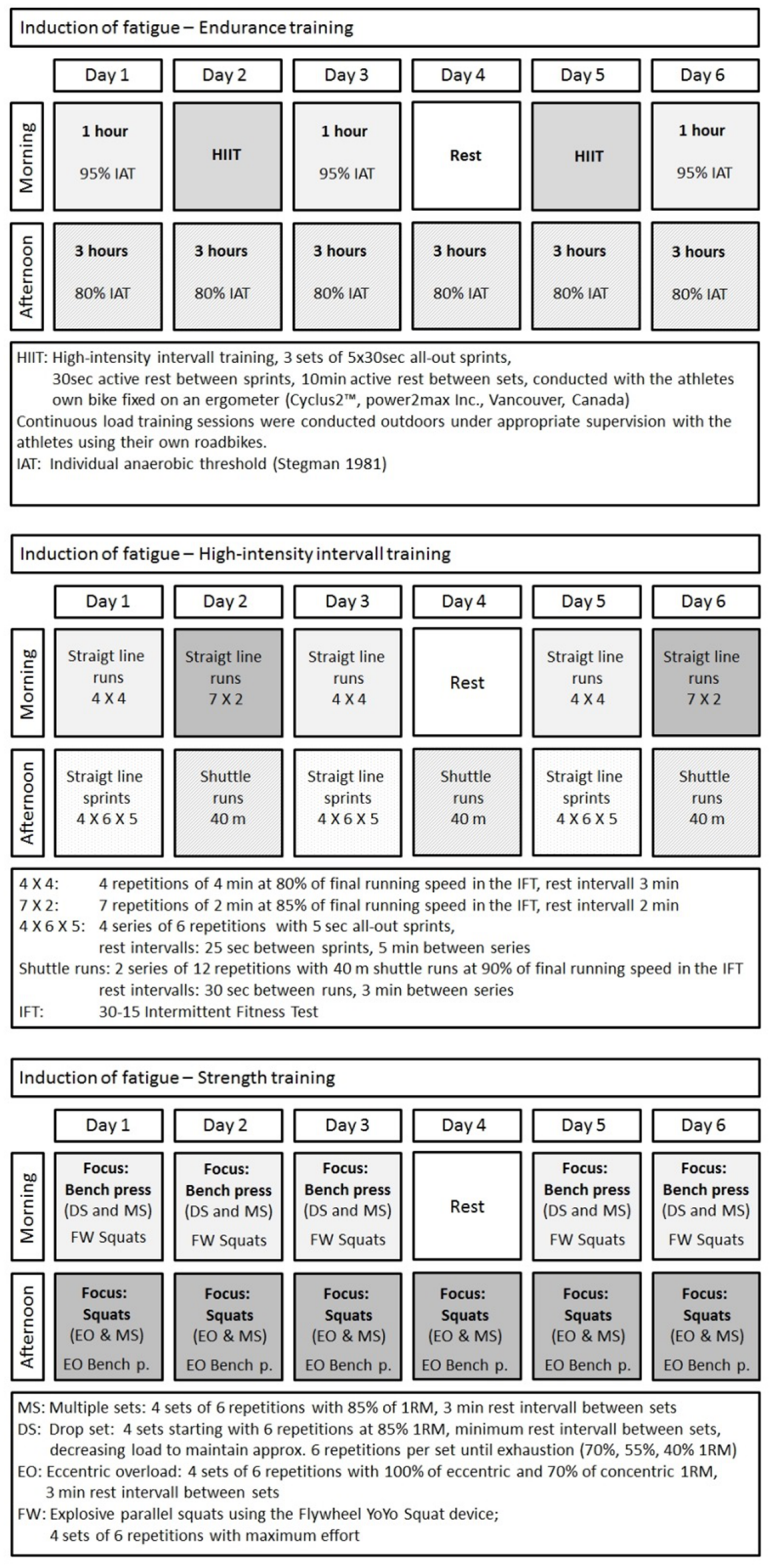
Schedules of discipline specific simulated training camps.

The 6 day training intervention comprised 11 training sessions and was designed to induce a detectable decline in discipline specific performance (fatigue) while still being tolerated by well-trained athletes from the respective disciplines. Care was taken to implement a realistic, training-stage like design for every exercise mode. An overview over the training schedules for the three subgroups is provided in Fig 2.

### Statistical analysis

The Statistica 7 software package (StatSoft, Hamburg, Germany) was used for data analysis. Changes from day 1 to day 8 (Δfatigue) as well as from day 8 to day 11 (Δrecovery) were calculated for all parameters. Normal distribution could be verified for all outcome parameters with the exception of CRP and changes in discipline specific performance with strength training (Shapiro-Wilks test). The normally distributed variables are given as mean ± standard deviation if not otherwise specified. Differences between the 3 time points were tested using repeated measures analysis of variance (ANOVA; repeat factor: test; grouping factor: exercise mode) with post-hoc Scheffé-test were appropriate. Associations between changes in normally distributed parameters were tested using Pearson product moment correlation. CRP and changes in maximum isometric contraction force are provided as median (first; third quartile). Differences between time points were tested using Friedman ANOVA with post-hoc Wilcoxon test were appropriate. The significance level for the α-error was set at p < 0.05 for all tests.

### Rules for interpretation

The course of a blood-born parameter is considered to reflect changes in fatigue status if a reversible, fatigue dependent deviation from baseline is present (values from day 8 differ significantly from day 1 as well as from day 11). Only parameters which fulfilled this criterion were included in the correlation analysis. Furthermore, non-overlap of 95% confidence intervals for values from day 8 with days 1 and 11, respectively, is considered to indicate diagnostic value of individual measurements.

## Results

### Subjects

General subject characteristics for the 3 subgroups are presented in Table 2. With the exception of 1 minor training related injury no adverse events related to the testing and training procedures were observed.

**Table 2:** Subject characteristics.

	Cycling	HIIT	Strength	p
n	28 (♂: 23; ♀: 5)	22 (♂: 11; ♀: 11)	23 (♂: 14; ♀:9)	0.05
Age (years)	29±7	23±3	24±2	**<0.01**
Height (cm)	178±7	177±8	175±8	0.22
Weight (kg)	72±9	70±7	72±11	0.67
VO_2peak_ (ml/kg/min)	58±8*	58±10^#^	53±8^#^	**0.03**
Max. workload	338±30 (W)	16.3±2.1 (km/h)	15.7±1.7 (km/h)	**n.a.**

Means ± standard deviation; VO_2peak_: Peak oxygen uptake

*: Stepwise incremental cycling test

^#^: Stepwise incremental treadmill test

^§^: for sex ratio

### Performance

For all 3 exercise modes a significant decline in discipline specific performance over the simulated training camp could be confirmed (cycling: longer time trial time p = 0.004, HIIT: lower mean peak velocity during repeated sprints p<0.001; strength training: lower maximum isometric contraction force p = 0.048). The restoration of performance over the recovery period was significant for cycling (p<0.001) and HIIT (p = 0.024) but failed to reach statistical significance for the strength training group (p = 0.094). Performance data for the male and female subgroups are provided as supplemental material (S1 Table).

### Courses of blood-born parameters

With the expectable exception of free-testosterone concentrations, no differences between males and females could be observed for the courses of blood-borne parameters. Therefore, data were pooled over sex for further analysis.

**Fig 3:**
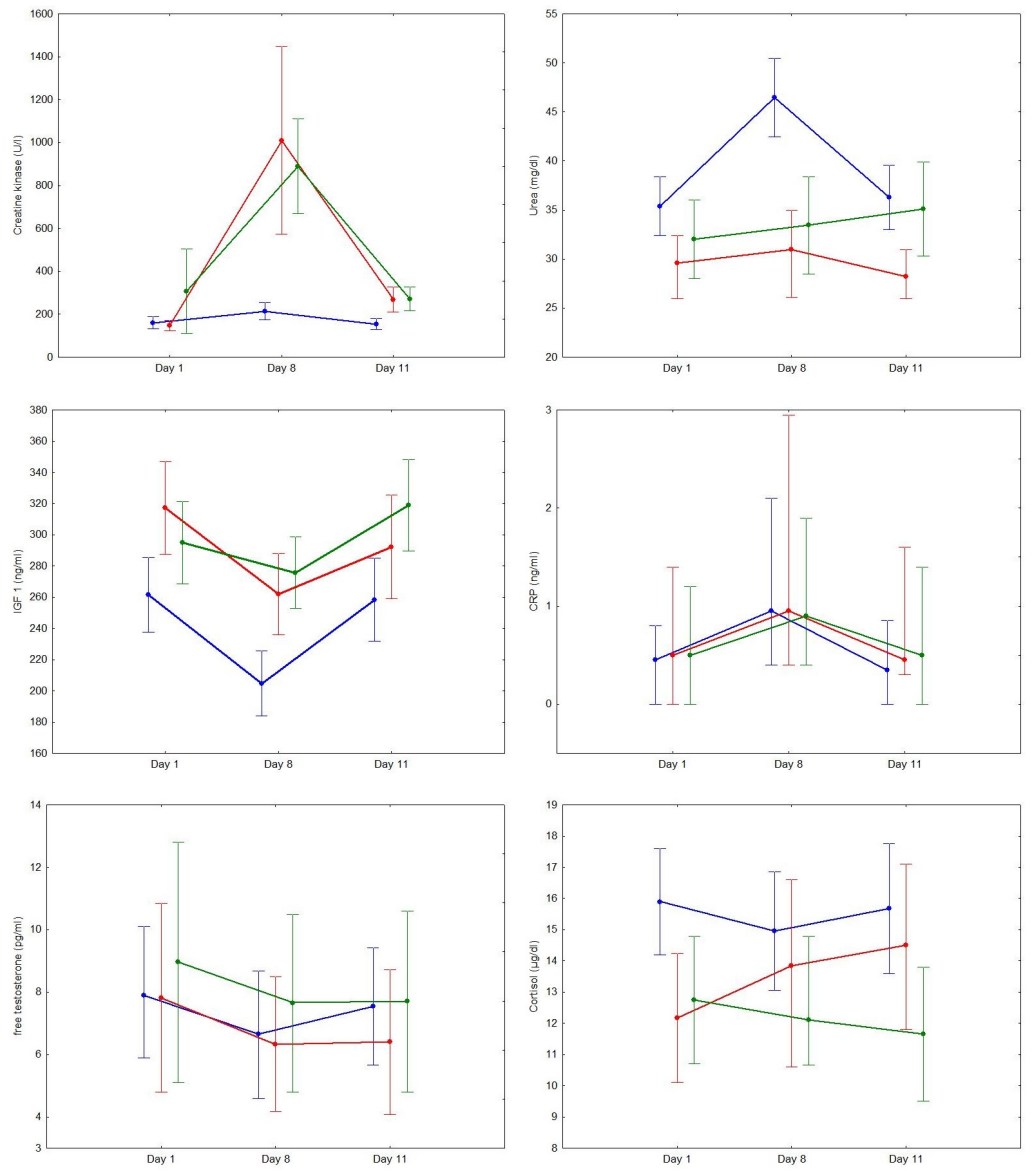
Courses of selected blood-born parameters. Means ± 95% confidence intervals (for CRP: median and interquartile range). Blue: cyclists; red: ballgame players; green: strength athletes.

For fatiguing endurance training, reversible deviations from baseline could be demonstrated for CK, urea, CRP, free-testosterone, IGF-1 as well as for the ratios of free-testosterone/cortisol and ACTH/cortisol. For urea and IGF-1, 95% confidence intervals from day 8 did not overlap with values from day 1 and day 11 (Fig 3). For HIIT and strength training, CK showed a fatigue-dependent time course with separated 95% confidence intervals. For strength training, the time course of CRP was also fatigue-dependent. Discipline-specific variations as Δfatigue and Δrecovery for all blood-borne parameters are summarized in Table 3. Measured values are provided as supplemental material (S2 Table).

**Table 3:** Changes in blood-born indicators of fatigue and recovery.

	Endurance		HIIT		Strength	
	Fatigue	Recovery	Fatigue	Recovery	Fatigue	Recovery
ΔCK [U/l]	**54 ± 84**	**-60 ± 83**	**863 ± 952**	**-741 ±842**	**582 ± 649**	**-618 ±419**
p	**0.002**	**<0.001**	**-0.001**	**<0.001**	**<0.001**	**<0.001**
Δ Urea [mg/dl]	**11 ± 9**	**-10 ± 10**	2 ± 6	-2 ± 7	1 ± 9	2 ± 8
p	**<0.001**	**<0.001**	n.a.	n.a.	n.a.	n.a.
Δ CRP [ng/ml]p	**0.6**	**-0.6**	**0.4**	**-0.4**	**0.4**	**-0.3**
	**(0.0; 1.5)**	**(1.5; -0.3)**	**(0.0; 2.0)**	**(-1.4; 0.0)**	**(0.0; 0.8)**	**(-0.8; 0.0)**
	**<0.001**	**<0.001**	**0.080**	**0.006**	**0.003**	**0.003**
Δ f-T [pg/ml]	**-1.3 ± 2.1**	**0.8 ± 1.5**	-1.5 ± 2.0	0.1 ± 2.0	-1.3 ± 4.2	0.0 ± 1.5
p	**<0.001**	**0.038**	0.027	0.964	n.a.	n.a.
Δ Cortisol [μg/dl]	-0.9 ± 3.7	0.7 ± 4.2	1.3 ± 4.3	0.9 ± 5.1	-0.6 ± 4.9	-0.4 ± 4.7
p	n.a.	n.a.	n.a.	n.a.	n.a.	n.a.
Δ fT/Cortisol [] *10^8^	**-0.1 ± 0.1**	**0.1 ± 0.3**	-0.1 ± 0.2	-0.1 ± 0.4	0.1 ± 0.5	0.0 ± 0.5
p	**0.037**	**0.009**	0.197	0.317	n.a.	n.a.
Δ Gln [μmol/l]	-1 ± 77	12 ± 64	50 ± 75	19 ± 83	80 ± 128	-53 ± 111
p	n.a.	n.a.	<0.05	0.979	0.008	0.105
Δ Glu [μmol/l]	4 ± 9	5 ± 11	-6 ± 9	6 ± 9	9 ± 14	10 ± 16
p	0.181	0.043	n.a.	n.a.	0.013	0.013
Δ Gln/Glu []	-4 ± 9	-2 ± 8	3 ± 4	-2 ± 4	-5 ± 9	-3 ± 4
p	0.081	0.575	0.021	0.082	0.030	0.223
Δ IL 6 [pg/ml]	0.4 ± 4.2	-0.2 ± 4.1	-0.3 ± 1.9	0.3 ± 0.8	0.7 ± 2.0	-0.2 ± 2.4
p	n.a.	n.a.	n.a.	n.a.	n.a.	n.a.
Δ HGH [ng/ml]	-0.9 ± 3.6	0.0 ± 6.3	0.4 ± 4.6	-1.2 ± 3.8	-2.4 ± 4.0	0.7 ± 3.1
p	n.a.	n.a.	n.a.	n.a.	0.019	0.696
Δ IGF 1 [ng/ml]	**-56 ± 28**	**53 ± 29**	-52 ± 73	25 ± 40	-19 ± 29	43 ± 55
p	**<0.001**	**<0.001**	0.002	0.116	0.197	<0.001
Δ IGF BP3 [μg/ml]	-0.1 ± 0.2	0.2 ± 0.2	-0.1 ±0.3	-0.1 ± 0.3	0.3 ± 0.2	-0.1 ± 0.4
p	0.156	<0.001	0.119	0.885	<0.001	0.680
Δ ACTH [pg/ml]	-7 ± 11	3 ± 6	-11± 7	6 ± 12	1 ± 7	0 ± 7
p	0.001	0.210	n.a.	n.a.	n.a.	n.a.
Δ TNF [pg/ml]	-0.1 ± 3.5	-0.6 ± 3.2	0.6 ± 6.0	-3.9 ± 6.2	-0.5 ± 5.4	2.3 ± 7.3
p	n.a.	n.a.	n.a.	n.a.	n.a.	n.a.
Δ ACTH/Cortisol [] *10^8^	**-0.4 ± 0.6**	**0.3 ± 0.4**	-0.7 ± 0.5	0.3 ± 0.7	0.1 ± 0.4	0.2 ± 0.4
p	**0.001**	**0.039**	n.a.	n.a.	n.a.	n.a.

Means ± Standard deviations (for CRP: median (interquartile range)); Fatigue: day 8 minus day 1; Recovery: day 11 minus day 8; Bold letters: Fatigue dependent course for this parameter and exercise mode; n.a.: No post-hoc test was conducted due to lack of significant global effect for the repeat factor; CK: creatine kinase; f-T: free testosterone; Gln: glutamine; Glu: glutamate; IL-6: interleukin 6; HGH: human growth hormone; IGF-1: insulin like growth factor 1; IGF-BP3: IGF binding protein 3; ACTH: adreno-corticotropic hormone; TNF: tumor necrosis factor

A significant test-by-discipline interaction, indicating different courses of the respective blood-borne markers between disciplines, was present for the following parameters: CK (p<0.001), urea (p<0.001), IGF-1 (p = 0.004), IGF-BP 3 (p<0.001), and ACTH (p = 0.010).

### Association between changes in blood-borne indicators and changes in discipline specific performance

No significant correlations could be found between changes in blood-born markers and changes in discipline specific performance (cycling: ΔCK fatigue p = 0.139 r = -0.29; Δ CK recovery p = 0.276 r = -0.21; Δurea fatigue p = 0.943 r = -0.01; Δurea recovery p = 0.982 r<0.01; ΔIGF-1 fatigue p = 0.330 r = -0.19; ΔIGF-1 recovery p = 0.928 r = 0.02; HIIT: ΔCK fatigue p = 0.200 r = 0.33; ΔCK recovery p = 0.621 r = -0.12; strength training: ΔCK fatigue p = 0.383 r = 0.19; ΔCK recovery p = 0.934 r = 0.18). This analysis was confined to fatigue-dependent parameters.

## Discussion

The main aim of the present study was to characterize changes in blood-borne indicators of fatigue induced by a period of standardized, discipline-specific training and the subsequent recovery period. In cyclists, representing high-volume endurance training, a reversible, fatigue dependent deviation from baseline was seen for urea, IGF-1, CK, free-testosterone and CRP. For urea and IGF-1, which both reflect the metabolic aspect of fatigue, 95% confidence intervals for fatigued and recovered time points did not overlap. For strength and HIIT training a reversible, fatigue-dependent deviation from baseline was seen for CK and CRP (strength training only). The magnitude of changes in CK, which reflect muscle fiber damage, was much higher with HIIT and strength training, respectively, compared to cycling (cp. Fig 3) and 95% confidence intervals for fatigued and recovered time points did not overlap for these subgroups.

The subgroup-dependent sets of blood-borne indicators for which a fatigue-dependent time course could be confirmed plausibly reflect the physical strain characterizing the respective training modes. Overload training in cyclists is mainly associated with a marked increase in caloric expenditure, temporal depletion of carbohydrate stores and subsequent protein breakdown for gluconeogenesis and ATP production [12]. These characteristics are reflected by an increase in urea and a decrease in IGF-1 in the fatigued state (day 8). Urea, the excreted form of nitrogen in humans, is a classic fatigue marker in endurance disciplines indicating protein breakdown [2,5,11]. In the absence of excessive protein intake, which could be ruled out by nutritional protocols in this trial, serum amino acids as well as proteins in serum and skeletal muscle are the most likely nitrogen sources. Insulin-like growth factor 1 (IGF-1) is a messenger substance produced in the liver which transmits the effects of human growth hormone (HGH) to peripheral tissues and mimics the pro-anabolic and blood-glucose lowering effects of insulin. A decrease in IGF-1 accompanied by an increase in the respective binding protein (IGF-BP3) has been proposed to indicate a state of glucose austerity after depletion of carbohydrate stores due to endurance training [4]. In the current study, IGF-1 showed the most pronounced fatigue-dependent time course in endurance athletes whilst no significant changes in IGF-BP3 could be observed.

Strength training and HIIT in running are characterized by a considerable proportion of eccentric force production and high muscular tension. This leads to muscle fiber damage resulting in leakage of the enzyme creatine kinase (CK) from the sarcoplasm into the blood stream. This effect is known for a long time and makes CK a classical blood-borne marker of strain and fatigue in the relevant disciplines [2,5,11]. The magnitude of changes in serum CK concentration for strength training and HIIT (about 3 to 7-fold) together with the ease and low cost of measurement make it tempting to consider CK as the decisive if not only blood fatigue indicator for these disciplines. However, it should be kept in mind that muscular damage is only one aspect of strain and fatigue and other aspects like vegetative balance, anabolic-catabolic balance or psychological alterations may play a role in the overall need for recovery.

Only previously proposed blood-borne indicators with a plausible physiological connection to exercise-induced fatigue were studied in this trial. Despite these criteria, the majority of parameters failed to show fatigue-dependent time courses, in particular for HIIT and strength training. In some cases, numerical differences in a direction consistent with previous reports and hypotheses were present but failed to reach statistical significance. This was the case for IGF-1, IGF-BP3 and HGH in strength-training as well as for IGF-1 in HIIT. As already stated above, IGF-1, IGF-BP3 and HGH are functionally connected and mainly represent the metabolic aspect of fatigue. A lesser involvement of the these processes in HIIT and strength training as compared to endurance training may therefore partly explain smaller effect sizes of fatigue-induced changes. Effect sizes for hormone levels were generally low (Table 3) despite standardized sampling conditions. It may be speculated that hormone responses to exercise stress tests [5,13] or pharmacological stimulation may have shown more pronounced fatigue dependent changes. However, such procedures are not suitable for routine application in healthy athletes and have therefore not been included in the present study.

Another picture emerges for the serum concentrations of the amino acids glutamine (Gln) and glutamate (Glu) of which the time courses mostly failed to resemble the expected pattern [3,14]. Lower Gln and higher Glu concentrations have already been reported more than 20 years ago in extreme forms of exercise-induced fatigue such as the overtraining syndrome [14]. More recently, a model of training tolerance based on the Gln/Glu ratio has been proposed [3]. Smith and colleagues [3] monitored Gln and Glu concentrations in relation to training load and performance in 52 elite athletes and reported lower Gln and higher Glu concentrations under heavy training load as compared to preseason values when athletes were relatively rested. In the present study, we could not confirm such fatigue-dependent and inverse time courses of Gln and Glu. One possible reason for these divergent findings concerns trial duration. The observational study by Smith et al. is much longer compared to the present experimental trial. The possible impact of this methodological difference is difficult to estimate because only little is known about the time course of fatigue and recovery related changes in serum Gln and Glu concentrations. However, parallel reductions in Gln and Glu after even shorter timeframes of fatigue induction (half-ironman triathlon [15]) support a possible relevance of the duration of fatigue development for changes in serum amino acids. Performance level and included disciplines of the athletes might also be considered. In this respect it is interesting, that the numerical changes in Glu concentration and Gln/Glu ratio in the HIIT training group resemble the expected pattern even though this was not significant. This is consistent with a potential mechanism inducing changes in Gln and Glu concentration caused by intensive training: the conversion of Gln to Glu by the enzyme glutaminase during the excretion of hydrogen ions by formation of ammonia [3]. Taken together, our data lend little support for a decisive role of Gln and Glu in tracking short-term changes in the fatigue status of competitive athletes.

From the number of cytokines and regulatory molecules which have been reported to be influenced by exercise training [4] Interleukin 6 (IL-6) and TNF have been included in the present study. For neither of them a fatigue dependent time course or even a significant global effect of the repeat factor (day 1, day 8, day 11) could be found. In the case of IL-6 the reason for this is probably the fast time course of serum concentrations [4] leading to a normalization overnight before blood sampling the next morning. An increase in TNF would have been expected in particular for HIIT and strength training as a result of inflammatory processes in response to muscle damage. However, despite marked increases in CK serum TNF concentrations remained unchanged.

In competitive athletes the ultimate aim of fatigue assessment is the individualized fine tuning of training recommendations to balance maximized training load and adaptation on the one hand side and sufficient recovery on the other. Therefore, the accurate characterization of the current fatigue status in an individual athlete is more important for the practical value of a fatigue marker than significant differences in means for recovered vs. fatigued time points. In the present trial, despite a run-in resting phase and a standardized induction of fatigue, inter-individual variability was high for measured values at the respective time points (S2 Table and S1 Fig) as well as for fatigue-induced changes (Table 3 and S1 Fig). A possible solution to this unfortunate characteristic are individualized reference ranges. This approach has already been validated for other biomarkers within the field of sports medicine [16], and is implemented in the Athlete Biological Passport [17]. However, individualized reference ranges require longitudinal data from the individual athlete and have not been tested in the field of fatigue assessment so far.

### Limitations

The design of this trial is based on discipline-specific, standardized training interventions aiming at the induction of an equally standardized degree of fatigue. This experimental approach is adequate for the characterization of fatigue-induced changes in blood-borne markers. However, it has to be kept in mind that standardized training does not necessarily imply standardized fatigue. Otherwise, assessment of fatigue would be dispensable if training load is known. Therefore, in an effort to verify the achieved degree of fatigue on an individual basis, discipline specific performance has been assessed as gold-standard. A reversible, fatigue-induced reduction in discipline specific performance was apparent for all exercise modes examined. However, the variability of fatigue-induced changes was large and no significant correlation with changes in blood-borne markers could be found. Potential explanations for this finding include methodological as well as biological aspects. Although reliability was a key aspect in choosing performance indicators [18,19,20,21], random variability in parameters of discipline-specific performance and their fatigue induced changes [22] does probably play a role. No estimate of within-subject variability has been obtained in this trial due to the unavoidable additional time commitment and testing burden for the subjects. Temporal overlap of training-induced performance decline (fatigue) and performance increase (training effect) may also contribute to the observed inter-individual variation in changes of discipline specific performance. Another limitation associated with the experimental approach is that only one (albeit standardized) point on the continuum from ‘completely recovered’ to ‘completely fatigued’ has been studied. Generalization or linear interpolation of results to more or less fatigued states may not be appropriate. Likewise, seasonal variations in training and competition may impact on kind and magnitude of (true) fatigue as well as on gold-standard and surrogate measures in a sport specific manner [23,24,25]. This limits the value of fixed reference ranges (e.g. confidence intervals displayed in Fig 3) and underlines the need for an individualized interpretation of observed values.

A main advantage of the present trial is the comparative assessment of fatigue markers for contrasting exercise modes within a homogeneous methodological framework. However, this is associated with challenges regarding the standardization sports-related subject characteristics and training interventions between groups. As only specifically trained athletes were eligible for the respective study arms, exact equivalence of sport-specific subject characteristics (e.g. competition level, level of physical performance, habitual training load) between subgroups is difficult to ensure or even to assess. In view of individual performance testing and relative training intensities, slight differences in these characteristics seem unlikely to impact relevantly on main outcomes. However, minor influences on between-group comparisons may not be ruled out.

Analogously to subject characteristics, stringent standardization of training load, strain and fatigue between exercise modes is not feasible. Therefore, comparability between groups is based on discipline specific training schedules at the upper limit of what may be implemented in real-world training camps. With regard to discipline specific performance, differences between criterion parameters also have to be taken into account. This becomes apparent when looking at the restoration of performance over the recovery period in the strength training group which, in contrast to the other groups, is incomplete and fails to reach statistical significance. Potential explanations for this discrepancy include physiological as well as statistical aspects. From the physiological perspective the substantial (ultra-) structural muscle damage with strength-training or HIIT (supported by the large increase in CK) may lead to more time-consuming repair processes as compared to endurance training. Together with the maximal muscle tension required during performance testing in the strength training group this may at least partly explain the incomplete return to baseline in these subjects. From the statistical perspective it has to be kept in mind that, in contrast to measures of discipline specific performance in the endurance and HIIT groups, maximum voluntary isometric contraction force (MVIC) was not normally distributed and therefore non-parametric test with inherently lower statistical power had to be applied.

## Conclusions

The most promising blood-borne indicators of fatigue are urea and IGF-1 for endurance training (cycling) and CK for HIIT (team sports) and strength training, respectively. However, even in these parameters, the interindividual variability of measured values and fatigue induced changes is considerable, pointing to the potential value of an individualized interpretation. Moreover, it has to be kept in mind that only one point on the recovery-fatigue continuum for the 3 exercise modes has been studied and generalization to other fatigue states or training regimes may not be justified.

## Supporting Information

S1 Fig Individual courses for selected blood born markers. (JPG)

S1 PDF Raw data. (PDF)

S1 Table Changes in discipline specific performance. (DOCX)

S2 Table Measured values for blood-borne markers. (DOCX)

S1 Text Detailed description of exercise testing procedures. (DOCX)
