# Supplementary figures and images for: Blood-Borne Markers of Fatigue in Competitive Athletes – Results from Simulated Training Camps
Source: PLoS One. 2016 Feb 18;11(2):e0148810. doi: 10.1371/journal.pone.0148810 (PMC4758695; doi:10.1371/journal.pone.0148810)

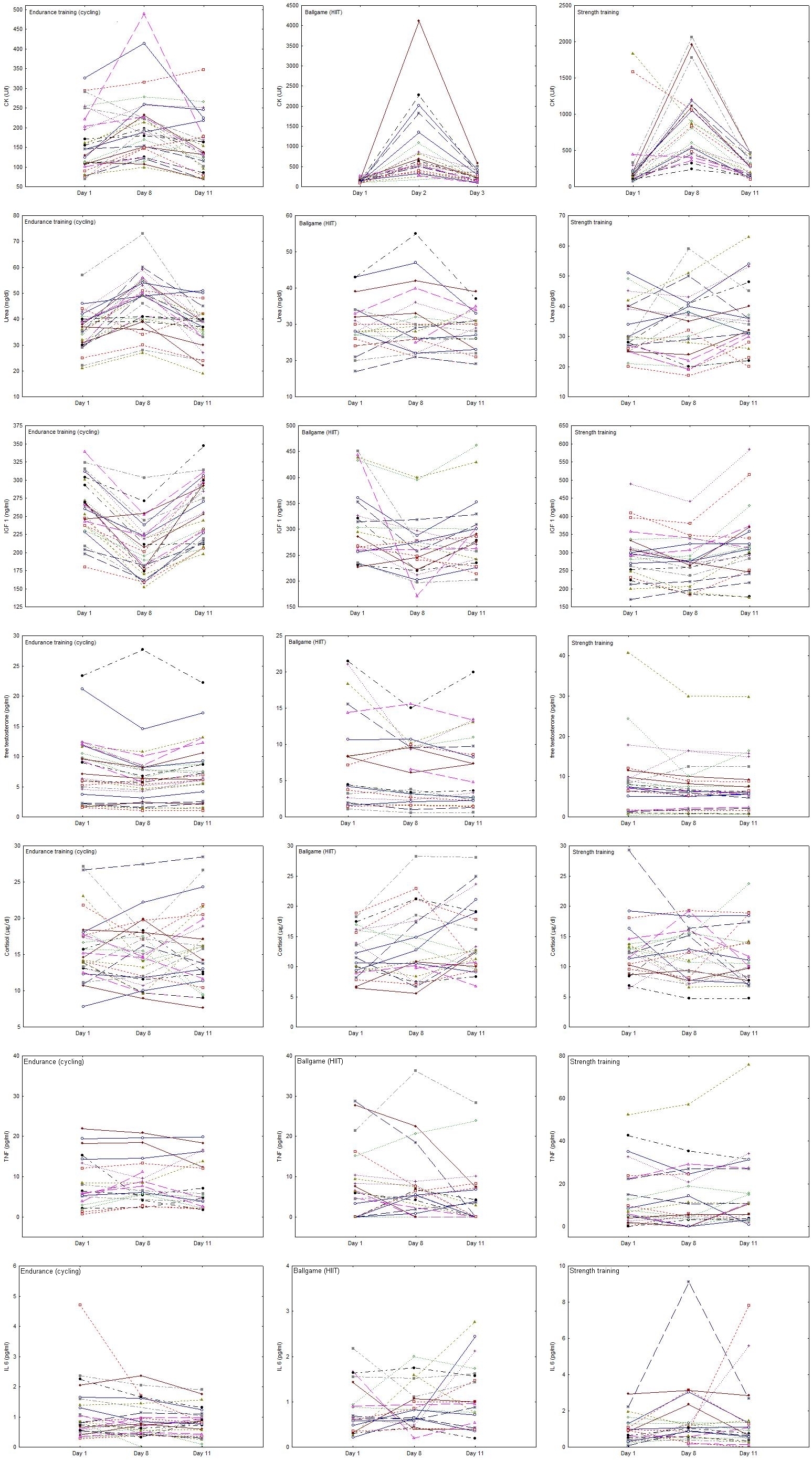

Supplement: S1 Fig — (JPG) [file pone.0148810.s001.jpg]
