# Supplementary material for: Blood-Borne Markers of Fatigue in Competitive Athletes – Results from Simulated Training Camps
Source: PLoS One. 2016 Feb 18;11(2):e0148810. doi: 10.1371/journal.pone.0148810 (PMC4758695; doi:10.1371/journal.pone.0148810)
Supplement: S1 PDF — (PDF) [file pone.0148810.s002.pdf]

	Sheet1																	
	1<br>Group 1: Endurance; 2: Team sports; 3: Strength	2<br>Sex 1: male; 2: female	3<br>Age (Years)	4<br>Height (cm)	5<br>Weight (kg)	6<br>VO_{2peak}<br>(ml/kg/min)	7<br>Day 1 - Baseline	8<br>CK (U/l)	9<br>Urea (mg/dl)	10<br>HGH (ng/ml)	11<br>IGF 1 (ng/ml)	12<br>IGF BP3 (µg/ml)	13<br>CRP (ng/ml)	14<br>TNF (pg/ml)	15<br>Free-testosterone (pg/ml)	16<br>Cortisol (µg/dl)	17<br>ACTH (pg/ml)	18<br>Glutamine (µmol/l)
1	1	1	22	178	70,9	59,80		145	46	3,168	228	2925	0,9	14,37	11,85	12,32	24,9	636
2	1	1	34	183	69,6			294	44	0,025	247	2058	0	0,63	5,2	14,06	5,2	753
3	1	1	25	173	69,1	62,70		255	34	0,916	237	2400	0	2,97	9,82	18,25	32,4	616
4	1	1	19	176	60,4	67,30		101	30	0,855	339	3085	0	5,76	9,18	12,54	26,5	705
5	1	1	23	174	60,4	65,60		108	40	0,058	304	2691	0	2,12	5,91	13,88	22,1	588
6	1	1	34	187	76,9	70,00		291	57	1,994	209	2172	0,2	5,11	4,94	11,14	18,4	643
7	1	1	25	187	72,5	47,60		108	38	0,46	267	1996	0,6	18,25	7,12	10,72	20,3	821
8	1	1	29	181	80,5	50,90		158	32	0,034	301	2694	0,2		6,18	13,92	13,1	636
9	1	1	27	179	71,2	55,50		195	37	0,099	264	2722	2,2	13,29	6,32	17,39	21,5	582
10	1	1	45	184	92,9	47,00		77	29	0,048	198	2824	0,7		2,19	10,88	24,7	568
11	1	1	30	189	79,3	62,60		124	39	0,044	312	3305	1,3	19,41	21,15	18,04	13,8	677
12	1	1	37	172	64,3	75,70		74	35	0,019	180	2512	2,2	12,05	5,93	17,59	17,8	595
13	1	1	42	171	82,0			113	41	0,379	272	2740	0,5		10,53	15,74	16,2	629
14	1	1	26	184	80,7	53,30		221	30	0,039	267	2194	0,4	3,89	11,94	17,79	25,6	725
15	1	1	24	190	76,3	53,70		155	39	0,486	293	2337	1,1	6,37	23,38	15,7	24,6	561
16	1	2	25	168	56,7	51,90		250	22	10,417	324	3096	0,5	8,05	1,92	27,13	12,2	602
17	1	2	26	165	59,2	54,10		111	31	16,507	270	2903	0,7		1,69	18,41	33,8	616
18	1	2	38	183	70,4	44,30		80	21	15,164	237	2893	2,5		1,8	23,07	7,9	609
19	1	1	20	177	65,5	62,00		254	43	0,294	314	2455	0		4,57	13,23	9,6	561
20	1	2	33	172	57,0	55,70		146	35	10,359	204	3929	0,4		2,25	26,67	22,7	465
21	1	1	38	185	79,2	63,00		326	42	4,478	260	2486	0	5,26	3,72	7,82	15,6	794
22	1	2	23	172	62,3	55,80		90	25	0,964	237	2953	0,2	1,3	1,56	21,77	49,0	554
23	1	1	24	175	65,0	61,70		111	30	0,04	230	2623	0	1,87	12,36	16,64	36,4	602
24	1	1	25	178	75,9	57,00		203	39	0,84	243	2233	0	5,99	12,36	15,22	39,3	718
25	1	1	23	176	73,8	65,40		171	30	4,421	269	2877	0	15,29	8,97	13,09	10,9	718
26	1	1	24	186	80,2	54,00		70	29	0,052	315	2764	2,7		9,7	13,28	8,8	684
27	1	1	24	182	72,8	62,67		129	37	0,035	246	2564	0,6	21,94	9,56	14,58	26,0	698
28	1	1	43	178	77,3	49,22		135	36	0,02	253	2225	0,5	8,49	11,65	14,21	10,5	835
29	2	2	27	168	60,2	63,20		158	34	3,091	234	2801	0	0	4,22	10,58	11,6	616
30	2	2	21	170	58,0	48,90		224	26				8,4		3,72	15,64		588
31	2	2	22	171	69,0	48,70		96	27	4,859	433	2837	0,6	15,12	4,4	16,03	22,5	588
32	2	1	26	186	81,0	67,50				9,423	444	3281		6,55			16,7	
33	2	2	28	169	57,0	54,50		125	24	0,925	231	2404	0	0	4,44	9,99	28,4	506
34	2	2	20	162	67,0	48,00		93	20	6,363	235	2982	1	21,47	3,21	18,21	6,8	664
35	2	1	23	180	66,0	60,60		200	39	0,121	227	2388	2,9	7,59	8,28	6,46	10,6	629
36	2	1	24	174	86,0	42,60		117	28		439	2751	0,5	9,48	18,41	10,03		520
37	2	1	23	182	66,0	70,90		111	28	0,677	268	2917	0,2	10,4	21,04	13,89	35,6	547
38	2	1	26	182	78,0	64,90		150	21	2,14	314	2386	1	0	15,54	11,45	20,7	595
39	2	1	20	171	71,0	66,50		99	43	11,9	361	2621	0	0	10,69	12,23	13,6	568
40	2	1	22	184	71,0	60,00		145	30	0,666	266	2559	0	0	7,16	7,8	4,7	718
41	2	1	22	188	72,0	81,90		112	27	0,042	303	1900	0,9	0	8,35	16,96	206,1	582
42	2	1	22	188	79,0	55,50		270	33	0,078	260	2058	0	4,64	14,41	8,94	13,6	623
43	2	1	23	183	70,0	75,60		138	43	0,069	321	2324	0,4	5,96	21,48	17,4	20,7	602

Sheet1

	19 Glutamate (µmol/l)	20 IL 6 (pg/ml)	21 Day 8 - Fatigue	22 CK (U/l)	23 Urea (mg/dl)	24 HGH (ng/ml)	25 IGF 1 (ng/ml)	26 IGF BP3 (µg/ml)	27 CRP (ng/ml)	28 TNF (pg/ml)	29 Free-testosterone (pg/ml)	30 Cortisol (µg/dl)	31 ACTH (pg/ml)	32 Glutamine (µmol/l)	33 Glutamate (µmol/l)	34 IL 6 (pg/ml)	35 Day 11 - Recovery	36 CK (U/l)	37 Urea (mg/dl)	38 HGH (ng/ml)
1	27	1,65		189	49	0,512	158	2697	1,5	14,58	8,20	11,85	9,7	636	20	1,61		218	51	0,194
2	27	0,83		315	34	1,406	175	1981	0,6	2,76	6,07	12,01	10,3	568	41	0,95		347	42	0,048
3	20	0,5		278	56	0,06	189	2422	0,0	5,76	6,43	14,09	12,8	657	34	0,59		266	35	0,021
4	20	1,04		125	41	0,447	252	2655	0,0	8,77	5,71	9,71	12,9	889	20	0,69		85	39	0,26
5	20	0,73		194	39	2,073	271	2519	0,0	2,33	5,63	11,04	23,6	588	20	0,62		97	35	0,05
6	27	1,07		224	73	4,096	162	2028	0,7	4,26	4,46	12,00	21,5	629	34	0,45		167	39	0,988
7	34	0,4		152	55	1,491	207	1893	0,9	18,47	6,38	8,93	11,7	780	34	0,73		132	39	0,033
8	54	1,4		225	40	0,058	219	2623	0,4		4,66	9,62	9,8	712	41	1,45		96	36	0,04
9	27	0,54		257	53	0,271	219	2594	3,9	9,51	5,18	15,08	10,7	732	34	0,87		251	27	0,109
10	41	0,8		122	50	0,258	162	2720	1,9		1,60	16,22	22,8	650	34	1,14		69	39	0,763
11	27	1,3		259	49	0,273	238	3185	0,4	19,62	14,54	22,20	15,5	616	34	0,83		245	37	0,088
12	14	4,71		147	51	0,128	159	2749	5,9	13,31	5,38	19,73	8,8	588	20	1,69		77	48	0,047
13	34	0,32		113	40	0,239	201	2929	5,0		7,87	15,51	6,6	657	27	0,42		179	40	0,294
14	34	0,6		489	56	0,084	182	2163	1,1	11,21	8,52	14,71	15,9	753	41	0,97		176	33	0,082
15	20	2,25		198	39	0,233	211	1881	1,0	5,43	27,70	18,32	12,3	629	27	1,66		135	37	0,038
16	27	2,36		222	28	8,222	303	3035	2,9	6,55	1,51	18,05	3,7	520	41	2,05		126	24	0,888
17	20	2,05		107	39	3,31	179	2700	1,3		2,45	18,04	27,6	602	20	2,36		69	22	33,758
18	48	0,34		99	27	6,784	170	2870	5,9		1,40	14,07	8,4	513	34	0,46		72	19	0,659
19	27	0,46		184	59	0,138	226	2680	0,0		4,20	10,70	6,2	629	27	0,43		165	39	0,079
20	20	0,54		153	60	5,677	182	3334	0,6	1,44	2,25	27,44	10,0	445	20	0,42		115	45	0,499
21	27	12,33		414	54	0,323	223	2455	0,0	6,08	3,11	10,04	8,6	664	48	18,87		224	50	0,184
22	27	0,28		149	30	8,772	201	3293	1,2	2,62	1,01	17,05	1,9	554	34	0,38		177	24	5,285
23	20	0,84		169	55	0,182	195	2603	0,6	5,62	8,44	17,51	15,3	582	34	0,01		119	34	0,062
24	34	0,34		227	50	0,17	223	2363	0,5	7,68	10,11	14,55	23,6	657	41	0,48		133	33	0,064
25	27	0,55		179	41	1,164	174	2652	0,0	4,09	6,75	11,55	10,9	705	27	0,33		163	40	1,386
26	27	1,6		195	46	0,124	244	2521	5,1		7,78	17,17	11,9	698	27			102	33	0,105
27	34	0,67		232	36	0,175	254	2489	2,3	20,88	8,18	19,89	41,3	691	41	0,77		138	30	0,12
28	14	0,85		459	49	0,19	152	2085	1,4	8,49	10,83	13,24	9,9	828	34	0,55		195	42	0,072
29	61	0,48		326	26	4,783	202	2443	0,0	0,88	3,21	10,52	9,3	575	34	0,82		108	27	0,47
30	41			356	21	8,349	241	2766	11,8	4,94	2,72	21,17	5,0	561	41	1		139	19	4,505
31	68	0,94		1088	32	6,63	395	3214	3,7	20,7	3,26	14,85	8,3	657	41	2		184	30	1,173
32		1,67		276	25	0,193	171	2551	0,0	0	6,59	9,78	11,4	636	20	0,2		98	35	0,166
33	41	0,3		634	26	1,304	220	1933	0,0	6,78	3,41	7,41	22,5	575	41	0,44		205	26	1,938
34	48	1,55		557	22	11,969	197	3253	1,4	36,28	3,78	28,26	9,0	636	48	1,52		117	22	2,554
35	48	1,43		686	42	0,3	245		1,6	0	6,12	5,54		657	61	0,4		226	39	0,032
36	88			820	28	3,794	400	3204	0,6	7,43	10,20	8,37	11,4	623	68	1,59		204	34	1,304
37	41	0,66		850	36	0,081	212	2334	0,4	8,82	9,33	7,28	19,5	554	61	0,48		422	31	0,921
38	68	0,62		499	29	0,27	318	2117	0,4	1,88	9,59	6,64	13,8	554	48	0,65		176	31	1,185
39	61	0,21		2021	47	1,805	288		0,5	5,5	10,73	14,84	17,9	643	68	0,64		412	33	0,18
40	48	0,6		404	30	4,487	277	2462	0,6	6,41	9,95	7,01	10,1	739	41	0,85		159	30	2,092
41	41	0,88																250	26	0,053
42	41	0,9		524	40				0,0		15,62	10,15		732	27			158	34	3,057
43	41	1,64		2276	55	0,125	220	2114	1,2	4,19	15,03	21,19	26,8	814	48	1,75		330	37	0,095

	Sheet1									
	39 IGF 1 (ng/ml)	40 IGF 1 BP3 (µg/ml)	41 CRP (ng/ml)	42 TNF (pg/ml)	43 Free-testosterone (pg/ml)	44 Cortisol (µg/dl)	45 ACTH (pg/ml)	46 Glutamine (µmol/l)	47 Glutamate (µmol/l)	48 IL 6 (pg/ml)
1	227	2824	0,9	16,31	9,25	12,99	17,1	664	34	1,23
2	232	1939	0,3	1,9	6,88	10,43	5,3	561	27	0,8
3	205	2445	0	2,15	8,9	16,19	17,9	712	34	0,09
4	311	2876	0,2	4,68	7,26	9,02	25,5	855	27	0,94
5	347	2964	0,2	4,68	6,32	12,64	24,2	684	34	0,73
6	219	2195	0,3	3,83	5,41	15,8	27,8	609	48	0,9
7	253	1985	0,5	12,35	6,16	7,63	17,3	718	34	0,85
8	244	3000	0		5,5	17,17	16,0	664	54	1,57
9	255	2620	2	16,63	6,02	18,92	25,9	671	20	0,79
10	216	3380	0,4		2,3	13,75	29,0	561	48	1,1
11	306	3641	0	19,83	17,22	24,34	20,9	629	48	0,76
12	206	2602	2,4	12,05	6,12	20,5	7,4	575	48	0,82
13	288	3197	0,9		7,55	9,34	11,0	725	48	0,31
14	230	2350	0,3		13,15	19,94	22,1	657	34	0,98
15	212	2097	0,4	7,12	22,21	12,59	15,2	677	41	1,31
16	314	3747	0,9	5,81	1,41	26,62	8,4	582	54	1,9
17	295	2993	0,4		2,12	17,14	20,3	588	20	1,75
18	198	3304	2,9		1,57	21,66	9,3	472	41	0,25
19	284	3051	0		6,37	14,19	7,9	650	34	0,4
20	213	3544	0,3	2,06	2,56	28,47	14,7	465	14	0,29
21	270	2569	0	3,89	4,14	11,35	24,5	855	61	15,98
22	303	3776	0,4	2,24	1,04	21,85	4,2	547	41	0,35
23	233	2778	0	5,06	8,71	9,48	8,1	684	48	0
24	298	2296	0	2,62	12,3	11,67	24,3	732	20	0,43
25	299	2861	0	1,75	8,67	12,29	14,8	753	34	0,89
26	275	2537	2,2		7,25	16,15	12,1	671	34	1,02
27	292	2472	0,8	18,35	10,57	14,24	32,3	739	41	0,6
28	208	2076	0,6	13,89	13,22	16,18	15,5	794	20	0,57
29	226	2337	0	3,85	2,71	9,07	11,9	664	34	0,71
30	228	2542	7,3	7,33	2,26	17,75	2,6	547	48	1
31	462	2867	1,2	23,86	2,56	12,1	12,0	664	68	1,73
32	303	2209	0	0	4,8	10,67	26,5	684	34	0,54
33	235	2176	0	4,21	3,59	8,32	21,7	643	54	0,19
34	202	2815	0,7	28,32	2,33	28,06	7,4	568	48	1,62
35	291	2354	0,3		7,33	12,47	21,6	746	68	0,4
36	430	2557	0,7	2,87	13,12	11,24	26,1	657	75	0,76
37	242	2493	0,3	10,13	13,42	13,31	17,9	513	88	2,12
38	329	2159	0,3	3,49	9,75	12,61	40,4	643	48	0,42
39	352	2422	0	6,78	8,2	18,98	58,1	671	61	0,35
40	286	2528	0,4	8,26	8,57	9,37	7,7	636	34	1,47
41	300	2254	0,4	0	10,95	9,5	20,2	691	41	0,77
42	263	2212	0	0	13,32	6,76	13,8	643	41	0,96
43	278	2120	0,5	0	19,94	19,11	26,7	602	48	1,57

	Sheet1																	
	1 Group 1: Endurance; 2: Team sports; 3: Strength	2 Sex 1: male; 2: female	3 Age (Years)	4 Height (cm)	5 Weight (kg)	6 VO_{2peak} (ml/kg/min)	7 Day 1 - Baseline	8 CK (U/l)	9 Urea (mg/dl)	10 HGH (ng/ml)	11 IGF 1 (ng/ml)	12 IGF BP3 (µg/ml)	13 CRP (ng/ml)	14 TNF (pg/ml)	15 Free-testosterone (pg/ml)	16 Cortisol (µg/dl)	17 ACTH (pg/ml)	18 Glutamine (µmol/l)
44	2	2	19	177	67,0	49,90		207	34	0,15	451	2948	1,7	4,49	1,09	13,49	8,1	417
45	2	1	21	180	72,0	66,40		178	32	1,689	286	2069	0	27,73	8,41	6,68	10,9	602
46	2	2	26	173	67,0	58,30		115	28	0,268	295	2513	0	6,41	1,8	9,15	8,4	554
47	2	2	25	184	74,0	48,80		233	31	1,204	326	3092	7,6	8,26	2,65	16,1	5,4	383
48	2	2	20	173	62,0	50,00		116	17	2,378	352	2724	2,8	28,7	1,96	8,18	16,2	568
49	2	2	24	176	72,0	50,10		118	28	7,681	256	2564	0,3	3,31	1,57	9,36	12,2	588
50	2	2	19	165	64,0	53,90		85	24	9,366	267	2597	1,4	16,21	1,43	18,84	12,5	568
51	3	2	27	177	76,0	47,90		144	30	7,527	297	2768	1,5	8,45	5,16	19,19	13,7	671
52	3	2	26	165	58,0	41,70		152	25	12,194	231	3298	4,8	0	1,64	18,04	5,2	479
53	3	2	24	167	58,0	45,90		68	21	11,337	281	2234	1,1	7,7	0,44	13,71	2,3	746
54	3	2	24	167	58,0	48,10		123	25	11,178	358	2611	0	5,86	7,02	14,61	32,3	657
55	3	1	21	172	71,0	61,00		153	28	0,104	253	2498	0	0	8,15	6,8	9,2	588
56	3	1	24	178	69,0	49,50		120	25	4,088	290	2352	0	1,36	9,06	8,82		725
57	3	1	22	182	72,0	60,10		217	25	9,682	308	2422	0	1,88	11,54	8,63	14,3	541
58	3	1	26	182	87,0	51,00		1840	42	0,722	200	1814	1,3	52,55	40,83	13,71	25,0	664
59	3	1	21	182	77,0	49,50		231	45	0,04	489	2105	0,4	32,61	17,83	12,54	23,0	554
60	3	1	24	184	82,0	67,10		181	40	0,033	212	2063	0	14,91	6,34	12,2	24,1	616
61	3	1	26	190	85,0	54,50		100	51	0,024	270	2081	0	5,12	7,43	16,31	24,7	766
62	3	2	24	161	58,0	43,00		80	20	2,351	396	2579	1	9,57	12,1	9,56	7,8	588
63	3	1	21	177	77,0	61,90		91	29	0,087	313	2444	0	12,57	8,02	13	15,2	773
64	3	2	21	167	67,0	39,30		445	27	6,628	292	3102	1,4	22,31	1,42	11,5	3,8	465
65	3	2	24	165	59,0	52,45		103	28	0,106	223	2408	1,2	42,62	1,08	8,38	11,7	486
66	3	1	26	172	67,0	60,40		329	29	0,158	262	2133	0	4,89	8,41	12,34	15,7	568
67	3	1	21	184	80,0	62,20		177	40	7,876	333	2491	0,3	3,97	9,81	10,23	20,8	568
68	3	2	24	164	61,0	40,70		136	30	0,389	248	2154	0,9	6,94	0,86	13,31	14,4	547
69	3	1	23	180	89,0	57,20		292	39	0,028	312	2082	0,7	2,69	9,59	6,4	13,5	561
70	3	2	28	163	52,0	47,30		79	27	0,375	170	2748	2,8	22,21	1,3	29,25	8,1	452
71	3	1	25	185	83,0	55,10		217	34	0,03	299	2184	0,5	35,06	7,37	11,33	18,8	718
72	3	1	22	180	81,0	58,30		1582	26	0,052	409	2110	0,3	23,83	6,41	10,42	16,8	561
73	3	1	25	175	84,0	55,90		200	49	1,314	336	1723	0,5	4,89	24,36	12,86	18,0	561

Sheet1

	19 Glutamate (µmol/l)	20 IL 6 (pg/ml)	21 Day 8 - Fatigue	22 CK (U/l)	23 Urea (mg/dl)	24 HGH (ng/ml)	25 IGF 1 (ng/ml)	26 IGF BP3 (µg/ml)	27 CRP (ng/ml)	28 TNF (pg/ml)	29 Free-testosterone (pg/ml)	30 Cortisol (µg/dl)	31 ACTH (pg/ml)	32 Glutamine (µmol/l)	33 Glutamate (µmol/l)	34 IL 6 (pg/ml)	35 Day 11 - Recovery	36 CK (U/l)	37 Urea (mg/dl)	38 HGH (ng/ml)
44	48	2,18		517	30	4,511	275	2667	3,7	5,31	0,55	18,52	5,7	424	48	1,11		506	28	3,985
45	54	0,32		4114	33	4,033	222	2063	1,4	22,49	9,55	10,80	20,9	657	34	1,07		579	23	0,188
46	48	0,27																376	31	0,163
47	82	9,8		497	22	5,693	297	2977	2,5	7,88	2,34	17,55	3,6	424	48	2,25		339	29	11,128
48	41	0,69		1824	21	2,378	257	2457	8,4	18,47	1,05	17,29	15,7	527	27	0,6		379	19	4,858
49	27	0,58		1346	22	5,331	275	2471	3,4	5,49	2,10	12,72	11,3	773	34	0,58		295	23	2,909
50	41	0,35		585	26	2,381	248	2440	0,7	7,59	1,60	22,89	10,4	705	54	0,41		258	21	9,509
51	27	1,31		1190	40	0,391	276	2636	1,4	14,25	5,10	18,33	28,9	718	48	3,04		465	36	0,337
52	61	1,08		839	19	1,382	183	3733	9,7	5,97	1,60	19,31	7,5	718	61	3,15		293	28	8,045
53	20	0,23		603	20	10,125	290	2704	2,2	4,45	0,78	15,06	4,4	554	34	0,46		151	22	0,701
54	14	0,61		363	19	0,732	340	3053	0,8	0	6,49	16,03	47,0	732	27	0,59		158	30	1,045
55	41	0,51		325	41	0,092	259	2463	0,0	3,11	6,50	4,73	10,5	609	41	0,56		123	48	0,035
56	20	0,4		1781	37	0,077	258	2624	0,8	0	6,90	7,06	11,7	718	41	1,27		296	34	1,252
57	41	0,87		1958	24	0,088	273	3051	0,0	0	10,09	9,40	10,4	657	48	2,36		469	32	1,173
58	27	1,96		876	51	0,207	206	2015	1,3	57,35	30,00	6,58	21,8	848	41	1,19		443	63	0,039
59	34			1199	43	0,113	440	2446	0,4	20,76	16,36	7,21	11,8	725	48	5		461	53	0,073
60	27	2,22		1110	50	0,126	219	2316	0,8	10,63	6,05	15,18	34,9	828	61	9,11		393	36	0,02
61	34	0,98		538	41	0,031	276	2238	0,2	0	6,27	7,75	16,3	698	41	1,06		117	54	0,362
62	27	0,95		480	17	0,408	380	2779	1,4	4,54	8,92	8,16	7,3	671	48	0,91		100	23	2,075
63	20	1,29		812	30	0,035	281	2817	0,0	18,82	7,57	10,74	14,1	787	48	1,35		164	37	0,427
64	20			405	22	1,064	307	3430	1,9	29,26	2,21	19,20	7,0	629	41	0,17		186	31	0,482
65	48	0,65		242	20	0,096	185	3198	3,7	35,38	0,74	16,23	7,2	629	54	1,05		156	22	5,793
66	34	0,4		2065	59	0,126	236	2133	0,5	5,02	12,52	9,17	10,4	814	48	0,39		468	45	4,145
67	48	2,94		1111	35	0,072	264	3001	0,6	5,5	8,26	7,66	9,7	773	48	3,14		270	40	0,047
68	41	0,3		546	28	0,204	189	2112	7,5	11,32	0,88	11,05	10,4	328	41	0,62		200	26	0,337
69	54	0,31		545	37	0,093	271	2512	1,3	4,07	16,44	12,47	15,2	623	68	1,25		276	35	1,119
70	54	0,08		453	29	3,162	196	3013	1,0	26,93	1,88	16,33	7,0	424	27	0,91		162	31	8,426
71	54	0,31		1049	38	0,057	324	2669	5,1	24,69	5,14	12,85	21,0	718	68	0,86		273	31	0,069
72	54	0,93		1058	32	0,85	347	2605	0,2	24,47	5,31	12,33	21,7	780	41	0,22		293	20	0,035
73	54	1,65		903	38	0,086	341	2072	0,9	3,35	10,08	15,61	22,9	705	48	1,34		323	31	0,085

	Sheet1									
	39 IGF 1 (ng/ml)	40 IGF 1 BP3 (µg/ml)	41 CRP (ng/ml)	42 TNF (pg/ml)	43 Free-testosterone (pg/ml)	44 Cortisol (µg/dl)	45 ACTH (pg/ml)	46 Glutamine (µmol/l)	47 Glutamate (µmol/l)	48 IL 6 (pg/ml)
44	257	2878	2,3	0	0,59	16,15	4,5	472	48	1,43
45	275	2018	0,4	7,44	7,35	9,98	21,4	739	41	1
46	242	2661	3	0	1,56	12,72	18,4	835	41	2,77
47	270	2966	3,5	0	3,33	23,59	6,2	506	54	4,22
48	309	2780	3,8	0	1,31	24,9	21,7	520	41	0,88
49	301	2757	1,6	0	2,25	21,12	23,7	855	48	2,44
50	214	2453	0,8	0	1,44	10,28	9,1	616	54	0,39
51	358	3039	0,9	0,81	5,66	18,43	27,1	643	54	1,32
52	251	4294	7,9	3,38	1,58	18,89	7,5	554	88	1,27
53	429	2424	2,1	14,95	0,69	23,73	8,4	561	41	0,53
54	313	2426	0	11,09	5,59	11,62	33,0	595	27	1,08
55	296	3028	0	3,67	6,45	4,73	13,2	609	41	0,36
56	320	2421	0,4	5,82	5,68	10,04	21,4	643	41	0,48
57	246	2361	0	10,52	9,27	7,64	8,6	643	34	0,65
58	302	2160	1,3	75,84	29,85	6,76	25,4	671	61	1,48
59	584	2529	0	34,14	14,91	8,35	22,9	623	48	1,67
60	240	2218	0	10,89	4,73	6,83	17,5	705	61	2,67
61	310	1862	0	3,24	6,11	7,2	11,4	691	48	1,09
62	515	3071	1,4		8,81	18,8	21,5	506	54	7,81
63	314	2200	0	15,49	7,6	10,54	20,6	691	41	0,91
64	373	3121	1,4	27,36	2,41	9,97	8,2	520	34	0,15
65	178	3013	2,7	31,47	0,73	7,66	5,3	629	75	0,75
66	283	2430	0,2	2,42	12,48	8,38	8,6	623	61	0,29
67	370	3116	0,4	5,67	7,45	9,73	15,6	677	41	2,85
68	176	2078	1,8	10,88	0,76	14,24	8,9	499	82	0,25
69	291	2313	8,9	1,84	15,74	8,21	14,9	486	82	5,58
70	216	3544	0,8	27,04	2,03	17,35	4,1	465	75	0,65
71	324	2190	1,3	31,24	5,74	11,07	26,0	985	54	0,58
72	340	2404	0		6,43	13,89	13,7	712	68	0
73	305	2042	0,5	2,59	16,38	13,89	23,9	746	68	1,37
