# Supplementary material for: Blood-Borne Markers of Fatigue in Competitive Athletes – Results from Simulated Training Camps
Source: PLoS One. 2016 Feb 18;11(2):e0148810. doi: 10.1371/journal.pone.0148810 (PMC4758695; doi:10.1371/journal.pone.0148810)
Supplement: S1 Table — (DOCX) [file pone.0148810.s003.docx]

| S1 Table: Changes in discipline specific performance | | | | | | | |
| --- | --- | --- | --- | --- | --- | --- | --- |
|  | Measured values | | | Changes with fatigue and recovery | | | |
|  | Day 1 | Day 8 | Day 11 | Δ Fatigue | p | Δ Recovery | p |
| Cycling  Time trial time [sec]  ♂ 40 km time trial | 3942 ± 212 | 4010 ± 201 | 3929 ± 219 | 68 ± 114  (2 ± 3 %) | 0.004  0.011 | -78 ± 108  (-2 ± 3 %) | <0.001  0.002 |
| ♀ 20 km time trial | 2123 ± 64 | 2169 ± 118 | 2124 ± 105 | 47 ± 85  (2 ± 4 %) | 0.235 | -46 ± 102  (2 ± 5 %) | 0.257 |
| HIIT  Mean peak velocity [m*s^-1^]  ♂ | 5.45 ± 0.29 | 5.33 ± 0.27 | 5.43 ± 0.25 | -0.11± 0.11  (-2 ± 2 %) | <0.001  0.007 | 0.09 ± 0.06  (2 ± 1 %) | 0.024  <0.001 |
| ♀ | 4.54 ± 0.19 | 4.34 ± 0.19 | 4.52 ± 0.38 | -0.20± 0.14  (-4 ± 3 %) | <0.001 | 0.18 ± 0.36  (4 ± 8 %) | 0.139 |
| Strength  MVIC [N]  ♂ | 1492 ± 341 | 1458 ± 276 | 1474 ± 295 | -41 (-106; 48)  (-3 (-7; 3) %) | 0.048  0.330 | 26 (-25; 62 )  (2 (-1; 5) %) | 0.094  0.300 |
| ♀ | 756 ± 88 | 722 ± 95 | 746 ± 98 | -40 (-51; -6)  (-6 (-6; -1) %) | 0.021 | 7 (0; 66)  (1 (0; 8) %) | 0.123 |
| Means ± Standard deviation or median (interquartile range) as appropriate  Mean velocity during repeated sprint tests  MVIC: Maximum voluntary isometric contraction force (mean of squad and bench press exercises)  Δ Fatigue: Change from day 1 to day 8; Δ Recovery: Change from day 8 to day 11 | | | | | | | |
