# Supplementary material for: Blood-Borne Markers of Fatigue in Competitive Athletes – Results from Simulated Training Camps
Source: PLoS One. 2016 Feb 18;11(2):e0148810. doi: 10.1371/journal.pone.0148810 (PMC4758695; doi:10.1371/journal.pone.0148810)
Supplement: S2 Table — (DOCX) [file pone.0148810.s004.docx]

| **S2 Table – Measured values of blood-born indicators** | | | | | | | | | |
| --- | --- | --- | --- | --- | --- | --- | --- | --- | --- |
|  | **Endurance (cycling)** | | | **Ballgame (HIIT)** | | | **Strength training** | | |
|  | **Day 1** | **Day 8** | **Day 11** | **Day 1** | **Day 8** | **Day 11** | **Day 1** | **Day 8** | **Day 11** |
| CK [U/l] | 161 ± 74 | 215±101 | 155 ± 69 | 147 ± 52 | 1010±933 | 269±134 | 307±454 | 889±510 | 271±126 |
| Urea [mg/dl] | 35 ± 8 | 46 ± 11 | 36 ± 8 | 29 ± 7 | 31 ± 9 | 29 ± 6 | 32 ± 9 | 33 ± 11 | 35 ± 11 |
| CRP [ng/ml] | 0.5 (0.0 I 0.8) | 1.0 (0.4 I 2.1) | 0.4 (0.0 I 0.9) | 0.5 (0.0 I 1.4) | 1.0 (0.4 I 3.0) | 0.5 (0.3 I 1.6) | 0.5 (0.0 I 1.2) | 0.9 (0.4 I 1.9) | 0.5 (0.0 I 1.4) |
| f-T [pg/ml] | 8.0 ± 5.4 | 6.7 ± 5.2 | 7.5 ± 4.9 | 7.8 ± 6.6 | 6.3 ± 4.6 | 6.4 ± 5.2 | 9.0 ± 8.9 | 7.7 ± 6.6 | 7.7 ± 6.7 |
| Cortisol [μg/dl] | 15.9 ± 4.5 | 15.0 ± 4.3 | 15.7 ± 5.5 | 12.3 ± 4.0 | 13.6 ± 6.4 | 14.5 ± 5.9 | 12.7 ± 4.8 | 12.1 ± 4.4 | 11.7 ± 4.9 |
| f-T/Cortisol [ ]*10^8^ | 0.54 ± 0.34 | 0.46 ± 0.30 | 0.56 ± 0.38 | 0.71 ± 0.59 | 0.64 ± 0.54 | 0.55 ± 0.50 | 0.80 ± 0.72 | 0.86 ± 1.00 | 0.86 ± 0.95 |
| Gln [μmol/l] | 650 ± 87 | 649 ± 95 | 661 ± 97 | 573 ± 74 | 623 ± 103 | 642 ± 99 | 602 ± 96 | 682 ± 122 | 629 ± 110 |
| Glu [μmol/l] | 28 ± 9 | 32 ± 8 | 37 ± 12 | 51 ± 15 | 45 ± 13 | 51 ± 14 | 37 ± 14 | 46 ± 11 | 56 ± 17 |
| Gln/Glu [ ] | 26 ± 10 | 22 ± 7 | 20 ± 8 | 12 ± 4 | 15 ± 6 | 13 ± 4 | 20 ± 11 | 15 ± 4 | 12 ± 4 |
| IL-6 [pg/ml] | 1.5 ± 2.3 | 1.5 ± 3.5 | 1.3 ± 2.9 | 1.3 ± 2.1 | 1.0 ± 0.6 | 1.3 ± 1.0 | 1.0 ± 0.7 | 1.7 ± 2.0 | 1.5 ± 1.8 |
| HGH [ng/ml] | 2.6 ± 4.7 | 1.7 ± 2.6 | 1.7 ± 6.4 | 3.2 ± 3.8 | 3.6 ± 3.2 | 2.4 ± 3.0 | 3.3 ± 4.4 | 0.9 ± 2.1 | 1.6 ± 2.5 |
| IGF-1 [ng/ml] | 261 ± 40 | 205 ± 38 | 258 ± 42 | 313 ± 74 | 261 ± 61 | 286 ± 64 | 295 ± 72 | 276 ± 66 | 319 ± 95 |
| IGF-BP3 [μg/ml] | 2.6 ± 0.4 | 2.6 ± 0.4 | 2.8 ± 0.2 | 2.6 ± 0.4 | 2.6 ± 0.4 | 2.5 ± 0.3 | 2.4 ± 0.4 | 2.7 ± 0.4 | 2.6 ± 0.6 |
| ACTH [pg/ml] | 21 ± 10 | 14 ± 8 | 17 ± 8 | 24 ± 44 | 13 ± 6 | 19 ± 13 | 15 ± 8 | 16 ± 10 | 16 ± 8 |
| ACTH/Cortisol [ ]*10^8^ | 1.4 ± 0.7 | 1.0 ± 0.6 | 1.3 ± 0.7 | 1.9 ± 2.6 | 1.2 ± 0.8 | 1.5 ± 0.9 | 1.3 ± 0.6 | 1.4 ± 0.8 | 1.6 ± 0.9 |
| TNF [pg/ml] | 8.6 ± 6.5 | 8.5 ± 5.8 | 7.9 ± 6.2 | 8.4 ± 8.8 | 9.0 ± 9.2 | 5.1 ± 7.8 | 14.0 ± 14.6 | 13.5 ± 14.4 | 15.7 ± 17.6 |
| Means ± standard deviations  CK: creatine kinase; f-T: free testosterone; Gln: glutamine; Glu: glutamate; IL-6: interleukin 6; HGH: human growth hormone; IGF-1: insulin like growth factor 1; IGF-BP3: IGF binding protein 3; ACTH: adreno corticotropic hormone; TNF: tumor necrosis factor | | | | | | | | | |
