# Supplementary material for: Blood-Borne Markers of Fatigue in Competitive Athletes – Results from Simulated Training Camps
Source: PLoS One. 2016 Feb 18;11(2):e0148810. doi: 10.1371/journal.pone.0148810 (PMC4758695; doi:10.1371/journal.pone.0148810)
Supplement: S1 Text — (DOCX) [file pone.0148810.s005.docx]

**S1 Text: Discipline specific exercise testing - Detailed information**

**Endurance training - Cycling**

Determination of peak oxygen uptake and individual anaerobic threshold

Stepwise incremental exercise tests were conducted with an initial workload of 100 Watt for males and 50 W for females. Workload was increased by 50 Watts every 3 min until volitional exhaustion. A MetaLyzer 3B (Cortex Biophysik, Leipzig, Germany) was used for continuous respiratory gas exchange measurements. The mean of the 3 highest consecutive readings during exercise was taken as estimate of VO_2peak_. Maximum heart rate (200 minus age [years]), maximum blood lactate concentration (8 mmol/l) and maximum respiratory quotient during exercise (1,1) were used as objective criteria of exhaustion. 2 out of 3 criteria had to be fulfilled which was the case in all tests. Capillary blood samples were collected at rest, at the end of every 3min step, at cessation of exercise as well as at min 1, 3, 5, 7, and 10 of the post-exercise period. Analysis was carried out using an enzymatic-amperometric system (Super GL, Greiner, Flacht, Germany). From the course of blood lactate concentrations individual anaerobic threshold (IAT) was calculated ([6](#_ENREF_6)).

Discipline specific performance

Performance in laboratory based cycling time trials were used as measure of discipline specific performance in cyclists. Time trial distance was 40 km for males and 20 km for females. Capillary blood samples for determination of blood lactate concentration were samples at every split time (4 km). Tests were conducted with the athletes own bike fixed on a cycle ergometer (Cyclus2™, power2max Inc., Vancouver, Canada). Time of day was standardized for each subject. Heart rate (RS800, Polar Electro, Helsinki, Finland) and respiratory gas exchange (MetaLyzer 3B and MetaMax 3, Cortex, Leipzig, Germany) were measured continuously. Capillary blood samples for the determination of blood lactate concentrations were taken from the hyperemized left earlobe. Samples were immediately hemolyzed and analysis carried out using an enzymatic-amperometric system (Super GL, Greiner, Flacht, Germany).

**High intensity interval training (HIIT) - Ballgame**

Determination of peak oxygen uptake

A stepwise incremental running test to exhaustion was conducted on a motorized treadmill (Woodway GmbH, Weil am Rhein, Germany) using the following parameters: Initial speed: 8 km/h, step increment: 2 km/h, step duration: 3 min. Respiratory gas exchange measurements and objective control of exhaustion were conducted as specified above.

Discipline specific performance

Repeated sprint ability (RSA) was chosen as measure of discipline specific performance for team sport athletes. RSA shows close association with competitive performance in these disciplines ([2](#_ENREF_2), [5](#_ENREF_5)) as well as good reproducibility ([1](#_ENREF_1), [3](#_ENREF_3)). RSA was tested on a non-motorized treadmill (Woodway GmbH, Weil am Rhein, Germany) as previously published ([3](#_ENREF_3)). The test consisted of 6 maximal sprints each with a duration of 4 s interspersed by 20 s of passive recovery. Vigorous verbal encouragement was given to all subjects. Mean peak velocity was calculated as measure of RSA.

**Strength training**

Determination of peak oxygen uptake

The same protocol as for the HIIT subgroup was employed.

Discipline specific performance

The mean of maximum isometric contraction force (MVIC [N]) for half squat and bench press exercise, respectively, was chosen as measure of discipline specific performance in strength athletes. MVIC was preferred over the dynamic 1 repetition maximum (1RM) here because of its superior reliability. MVIC was measured using a Kettler Profiline Multitrainer 7812-10 with analysis software (Kettler GmbH, Ense-Parsit, Germany). For half squat exercise subjects were positioned in shoulder wide stand with slight external foot rotation. The starting position of 90° knee angle was controlled using a goniometer. For bench press exercise subjects were in a supine position with their feet remaining on the floor, the barbell at the nipple line and their elbow flexed 90°. To execute the test, subjects were asked to produce a maximum isometric contraction over a time of 3 sec with slow force development. Each test was performed twice interspersed by a 2 min rest period. The mean of the two trials was calculated as estimate of MVIC for that exercise.

Subsequently, 1 RM was determined for training prescription using a smith rack machine with guided barbell (Technogym Multipower, TechnoGym GmbH, Neu-Isenburg, Germany). Positioning of the subjects was similar to MVIC measurements, with the exception of starting knee angle for squat exercise (parallel squat position with the upper line of the thigh musculature in a horizontal line). The maximum permitting the subject to execute 5 to10 repetitions was determined and 1 RM was estimated using the formula published by Brzycki ([4](#_ENREF_4)).

**References**

1. Hughes MG, Doherty M, Tong RJ, Reilly T, Cable NT. Reliability of repeated sprint exercise in non-motorised treadmill ergometry. *International journal of sports medicine*. 2006;27(11):900-4.

2. Impellizzeri FM, Rampinini E, Castagna C et al. Validity of a repeated-sprint test for football. *Int J Sports Med*. 2008;29(11):899-905.

3. Oliver JL, Williams CA, Armstrong N. Reliability of a field and laboratory test of repeated sprint ability. *Pediatric Exercise Science*. 2006;18(3):339.

4. PJ M, Foster C, (Editors), Champaign Iao. Physiological assessment of human fitness. *Human Kinetics* 2006.

5. Rampinini E, Bishop D, Marcora SM, Ferrari Bravo D, Sassi R, Impellizzeri FM. Validity of simple field tests as indicators of match-related physical performance in top-level professional soccer players. *Int J Sports Med*. 2007;28(3):228-35.

6. Stegmann H, Kindermann W, Schnabel A. Lactate kinetics and individual anaerobic threshold. *Int J Sports Med*. 1981;2(3):160-5.
